# Transcriptional control of a metabolic switch regulating cellular methylation reactions is part of a common response to stress in divergent bee species

**DOI:** 10.1242/jeb.246894

**Published:** 2024-06-10

**Authors:** Helen V. Kogan, Shannon G. Macleod, Nicole C. Rondeau, Joanna Raup-Collado, Victoria A. Cordero, David Rovnyak, Corey A. Marshalleck, Meghna Mallapan, Melissa E. Flores, Jonathan W. Snow

**Affiliations:** ^1^ Biology Department, Barnard College, New York, NY 10027, USA; ^2^ Department of Chemistry, Bucknell University, Lewisburg, PA 17837, USA

**Keywords:** Cellular stress response, Unfolded protein response, Methylation, Honey bee, Alfalfa leafcutting bee

## Abstract

Recent global declines in bee health have elevated the need for a more complete understanding of the cellular stress mechanisms employed by diverse bee species. We recently uncovered the biomarker *lethal (2) essential for life* [*l(2)efl*] genes as part of a shared transcriptional program in response to a number of cell stressors in the western honey bee (*Apis mellifera*). Here, we describe another shared stress-responsive gene, *glycine N-methyltransferase* (*Gnmt*), which is known as a key metabolic switch controlling cellular methylation reactions. We observed *Gnmt* induction by both abiotic and biotic stressors. We also found increased levels of the GNMT reaction product sarcosine in the midgut after stress, linking metabolic changes with the observed changes in gene regulation. Prior to this study, *Gnmt* upregulation had not been associated with cellular stress responses in other organisms. To determine whether this novel stress-responsive gene would behave similarly in other bee species, we first characterized the cellular response to endoplasmic reticulum (ER) stress in lab-reared adults of the solitary alfalfa leafcutting bee (*Megachile rotundata*) and compared this with age-matched honey bees. The novel stress gene *Gnmt* was induced in addition to a number of canonical gene targets induced in both bee species upon unfolded protein response (UPR) activation, suggesting that stress-induced regulation of cellular methylation reactions is a common feature of bees. Therefore, this study suggests that the honey bee can serve as an important model for bee biology more broadly, although studies on diverse bee species will be required to fully understand global declines in bee populations.

## Teaser

**Highlighted Article:** In divergent bee species, a metabolic switch regulating cellular methylation reactions is induced by abiotic and biotic stressors, potentially serving as a biomarker of stress and an important mechanism for responding to diverse stressors.

## INTRODUCTION

Pollination services provided by diverse bee species are critical for the health of both agricultural and ecological systems. In the face of global bee declines (Zattara and Aizen, 2021), characterization of the mechanisms used by diverse bee species to respond to stress will contribute to a more complete understanding of the determinants of pollinator health (López-Uribe et al., 2019). In the honey bee, mortality at the individual and colony levels is likely the result of multiple interacting stressors, including nutritional stress due to loss of appropriate forage, chemical poisoning from pesticides, changes to natural living conditions brought about through large-scale beekeeping practices, myriad environmental changes due to climate change, and infection by insect parasites and pathogenic microbes (Hristov et al., 2020; Steinhauer et al., 2018). In multiple species, including the model invertebrates *Drosophila melanogaster* and *Caenorhabditis elegans*, these same environmental factors are known to contribute to organismal disease by negatively impacting the cellular process of protein synthesis, folding and degradation (also known as proteostasis) (Powers and Balch, 2013)*.* As proteostasis is a likely interaction point for the stressors causing disease in honey bees, we have previously characterized a number of model cell stress responses in *Apis mellifera*, or the western honey bee. We identified triggers and responses for the honey bee unfolded protein response (UPR) of the endoplasmic reticulum (ER) (Adames et al., 2020; Johnston et al., 2016), which responds to proteostatic perturbation in this compartment, the honey bee integrated stress response (ISR) (Flores et al., 2021; Snow, 2020), which responds to amino acid deprivation or ribosome dysfunction, and the Heat Shock Response (HSR) (Bach et al., 2021; McKinstry et al., 2017), which responds to disruption of proteostasis in the cytoplasm. Using well-defined abiotic stressors to trigger these responses to perturbed proteostasis, we recently used an unbiased approach to identify novel genes altered in expression during induction of these proteostatic network responses in the honey bee using transcriptome profiling (RNASeq) (Adames et al., 2020; Bach et al., 2021; Flores et al., 2021). Among these genes, we described the induction of members of the *l(2)efl* family of small heat shock protein genes (Shih et al., 2021), which have been proposed as stress biomarkers (Cho et al., 2022; McAfee et al., 2020a,b; Shih et al., 2021) and antiviral genes (Brutscher et al., 2017; McMenamin et al., 2018) in honey bees. However, other potential common stress genes were not explored.

In addition to understanding how honey bees are affected by and respond to various stressors, there is great interest in illuminating the factors that influence the health of non-*Apis* bee species (López-Uribe et al., 2019). While new genome resources allow for comparison of ‘omics’-based studies between bee species (Trapp et al., 2017), functional studies to support hypotheses generated using ‘omics’ data are equally important (Grozinger and Zayed, 2020). Evidence suggests that studies using honey bees cannot take the place of research on other bee species because of the diverse life histories within this phylogenetic group (Wood et al., 2020). For example, sterile workers of the eusocial honey bee might be expected to have a cellular stress response that diverges significantly from that of solitary bees constrained by reproduction and somatic maintenance trade-offs. Although over 90% of all bee species are solitary (Danforth et al., 2019), the volume of functional studies using solitary bee species is far below that performed with honey bees and bumble bees, especially those focused on the cell and molecular level.

**Fig. 1.**
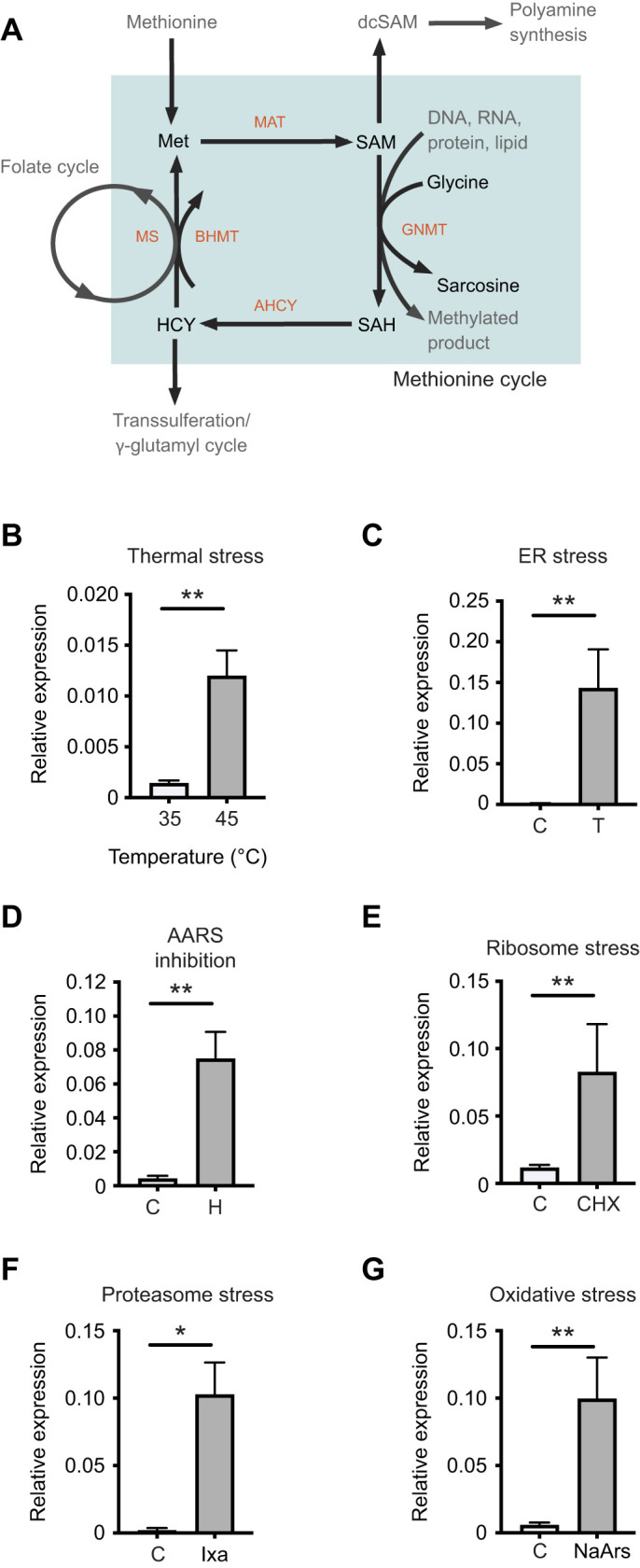
***Gnmt* is induced by diverse stressors in honey bees.** (A) Schematic diagram of the methionine cycle and the role of glycine N-methyltransferase (GNMT) in the cycle. MAT, methionine adenosyltransferase; dcSAM, decarboxylated *S*-adenosylmethionine; SAM, *S*-adenosylmethionine; SAH, *S*-adenosylhomocysteine; AHCY, adenosylhomocysteinase; BHMT, betaine-homocysteine methyltransferase; MS, methionine synthase. (B–G) Transcript levels of *Gnmt* relative to *β-actin* in midgut tissue from adult bees captured at the landing board after thermal stress [4 h at either 45°C (*n*=10) or 35°C (control temperature, *n*=7); B], or after pharmacological induction of endoplasmic reticulum (ER) stress (*n*=7 DMSO vehicle control, C; *n*=8 tunicamycin, T; C), aminoacyl tRNA synthetase (AARS) inhibition (*n*=8 DMSO vehicle control; *n*=8 halofuginone, H; D), ribosome stress (*n*=8 control, *n*=8 cycloheximide, CHX; E), proteasome inhibition (*n*=8 control, *n*=8 ixazomib, Ixa; F) or oxidative stress (*n*=8 control, *n*=8 sodium arsenite, NaArs; G). Data (means±s.e.m.) represent expression values of the genes of interest calculated using the 2^−Δ*C*_T_^ method for individual bees. Statistical significance is indicated by asterisks (*P<0.05, **P<0.01).

Here, we built on our work in honey bees by characterizing a novel target gene we hypothesized to be part of a common stress response in honey bees. We found that transcription of *glycine N-methyltransferase* (*Gnmt* for gene and mRNA versus GNMT for protein in accordance with *D. melanogaster* convention) is induced by both abiotic and biotic stressors. GNMT plays a key role in the methionine cycle (Fig. 1A), in which methionine is first converted to *S*-adenosylmethionine (SAM), and then *S*-adenosylhomocysteine (SAH), and finally homocysteine before being recycled back to methionine (Sanderson et al., 2019). GNMT is thought to be the main regulatory switch controlling SAM levels and the SAM/SAH ratio, and thus is considered to be a key metabolic switch controlling cellular methylation reactions. We then characterized the canonical UPR in a distantly related solitary bee species, the alfalfa leafcutting bee (*Megachile rotundata*) (Hedtke et al., 2013) and determined whether this putative ‘common’ stress response gene, *Gnmt*, is induced by the UPR in this species in order to compare stress responses in bee species with different life histories.

## MATERIALS AND METHODS

### Honey bee colonies and caging experiments

Honey bees were collected from outbred colonies in New York, NY, USA, consisting of a typical mix of *Apis mellifera* Linnaeus 1758 subspecies found in North America, at different times during the months of April–October. Source colonies were visually inspected for symptoms of common bacterial, fungal and viral diseases of honey bees. For experiments analyzing diverse stressors, adult bees were collected from the landing board and kept in 2.2×8.6×21.3 cm acrylic cages with a sliding door machined at Carleton Labs, Columbia University, USA. Cages were maintained in an incubator at 35°C in the presence of PseudoQueen (Contech, Victoria, BC, Canada) as a source of queen mandibular pheromone (QMP). For these experiments, bees were fed 33% sucrose via a modified 1.5 ml screw-cap tube, with sucrose solution alone or containing model stressors that are summarized in Table 1. These compounds and their effects in insects have been characterized in the model insect *D. melanogaster* (Chow et al., 2013; Deliu et al., 2017; Lundgren et al., 2005; Sykiotis and Bohmann, 2008) and honey bees (Adames et al., 2020; Flores et al., 2021; Johnston et al., 2016; Shih et al., 2021), with the exception of halofuginone, which has solely been used in honey bees (Flores et al., 2021; Snow, 2020), and ixazomib, for which the honey bee effects have not yet been published (J.W.S., unpublished observations). Specifically, we used 24 µmol l^−1^ tunicamycin to trigger the UPR, 200 µmol l^−1^ halofuginone to activate the ISR, 0.5 mg ml^−1^ cycloheximide to inhibit translation and 200 µmol l^−1^ ixazomib to perturb proteasome function. DMSO is the solvent for tunicamycin, halofuginone and ixazomib, so equivalent amounts of DMSO were added to the food of the control group for these treatments. For the model stressor of heat shock, which triggers the HSR, landing board bees were maintained for 4 h in cages at 35 or 45°C. We also investigated the impact of oxidative stress (induced by 0.001% sodium arsenite for 24–48 h). After treatment, midgut tissue [and additionally for thermal stress head tissue (predominantly brain and sensory organ tissue), thorax tissue (predominantly flight muscle) and abdominal wall tissue (predominantly fat body)] were dissected for gene expression analysis. Dissected tissues were immersed in RNAlater (Invitrogen, San Diego, CA, USA) for storage at −20°C prior to RNA extraction or flash frozen in liquid nitrogen for storage at −80°C for metabolite analysis. For UPR induction experiments with 7 day old honey bees, approximately 30 newly emerged bees were collected after hatching from a capped brood frame overnight in an incubator at 35°C (unless otherwise stated), added to the acrylic cages described above, and maintained in the same conditions as landing board bees above. At 7–9 days post-eclosion, honey bees were treated with tunicamycin in the same manner as alfalfa leafcutting bees below. All honey bee experiments were performed with a minimum of three independent trials, with similar results.

**Table 1.**
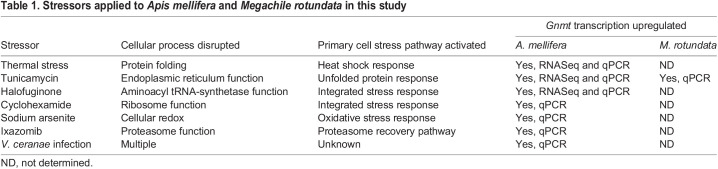
Stressors applied to *Apis mellifera* and *Megachile rotundata* in this study

### *Vairimorpha* (*Nosema*) *ceranae* infection of honey bees

A single biotic stressor, infection by the microsporidia parasite *Vairimorpha ceranae* was used. *Vairimorpha ceranae* spores were obtained from infected individuals from a colony and serially passaged through bees. To isolate spores for new infections, midguts from infected bees were individually crushed in 0.5 ml H_2_O with a pestle and spore number was assessed by light microscopy. Homogenized midguts were washed with water and resuspended in 1 mol l^−1^ sucrose solution. For experiments with newly eclosed bees*,* the soak method of inoculation was used as previously described (Parrella et al., 2024). Briefly, a 50 ml conical tube was filled to the 20 ml mark with newly eclosed bees (∼65 bees). Sucrose solution (1 ml) containing *V. ceranae* spores (2×10^6^ ml^−1^) was added to the conical tube, which was mixed by gentle inversion at 5 min intervals for a total of 30 min of exposure. Bees were then transferred to the cages described above and fed sucrose solution (and supplied with a ∼5 g pollen substitute patty) for the remainder of the experiment. At 9 days post-infection, honey bee midguts were dissected and placed in RNAlater (Invitrogen). To verify infection levels, qPCR using cDNA generated as detailed above was used to determine the relative amount of *V. ceranae β-actin* relative to honey bee *β-actin* levels.

### NMR metabolite quantification

Excised midguts were stored initially in individual micro-centrifuge tubes (−78°C, dry ice) for transport and subsequently stored at −80°C. A recently determined optimal aqueous metabolite extraction protocol for diverse bee samples (brain, head, body, whole bee) was used here (McDevitt et al., 2021). To each tube containing a midgut, 1 ml of a 2:1 v/v solution of acetonitrile/water was added along with two steel beads [1/8 inch and 1/16 inch (∼3.18 and 1.59 mm)]. A reciprocating bead-beating apparatus was used for thorough homogenization followed by high-speed centrifugation (10 min, 18,000 rcf) to pellet cell debris and precipitate biomacromolecules. The resulting supernatant was isolated and subjected to rotary vacuum centrifugation [3 h, 1 torr (∼133 Pa), no heating], resulting in small dry pellets that were frozen at −80°C. Each pellet was resuspended with vortex agitation in 350 μl of NMR buffer [99% D_2_O, 0.1 mmol l^−1^ TSP (IUPAC: sodium 3-trimethylsilylpropionate), 100 mmol l^−1^ phosphate, pH 7.4] and transferred to a Shigemi^TM^ susceptibility matched NMR tube. Because of small broad background (residual protein/lipid) signals, one-dimensional presaturation-CPMG (100 cycles, 1 ms interpulse delay) ^1^H NMR spectra were acquired [352 transients, 8 steady-state scans, 3 s presaturation, 4 s acquisition, 7 s recycle, *t*(π/2)=7.6 µs, 66 min each]. All spectra were obtained on a 14.1 T spectrometer (600 MHz for ^1^H, Varian Inc., Palo Alto, CA, USA; DDR1/VNMRS generation console, vnmrj 4.2) using an inverse triple resonance probe. Spectra were profiled manually by J.R.-C. and D.S.R. using the Chenomx NMR Suite 8.1 (Chenomx, Edmonton, AB, Canada) and are reported as concentrations (sample volume 0.350 μl). Full results and analyses of these data are forthcoming in another publication (N.C.R., J.R.-C., H.V.K., R. M. Cho, N. L. Lovinger, F. Wague, A. J. Loptakin, N. G. Texeira, M.E.F., D.R. and J.W.S., unpublished). Each sample was shimmed to an initial TSP line width of 0.8 Hz or lower. Analysis employed line broadening (0.3 Hz), phasing, baseline correction (spline), and reference deconvolution of the 0 ppm TSP peak.

### Alfalfa leafcutting bee pupa source storage and incubation conditions

Alfalfa leafcutting bee, *Megachile rotundata* (Fabricius 1787), prepupae in alfalfa leaf cocoons were acquired from a distributor (Crown Bees, Woodinville, WA, USA) and were maintained at 4°C until ready for use in caging experiments. For caging experiments, cocoons were placed in a single layer (approximately 100 cells per box) into 7×9×13 cm plastic boxes with loose fitting lids to allow for free air exchange and maintained in a 30°C incubator in complete darkness with a glass tray of water to ensure that appropriate humidity (typically 50–60%) was maintained. Because male alfalfa leafcutting bees typically eclose at 18–21 days when maintained at 30°C, 1.5 ml modified Eppendorf tubes with 30% sterile sucrose solution were placed into the containers at 18 days to provide a food source for any organisms that eclosed overnight. Eppendorf tubes were modified by piercing two opposing holes with a heated tack near the tip of the tube. After 18 days, the containers were checked 1–2 times daily. Males were removed immediately after eclosion to minimize chances of mating. Female alfalfa leafcutting bees typically eclose at 21–24 days when maintained at 30°C.

### Alfalfa leafcutting bee adult caging conditions and chemical treatments

Eclosed females were immediately transferred (4–6 per cage) to the acrylic cages described above, via InsectaVac Aspirator (Bioquip, Rancho Dominguez, CA, USA), and the incubators were maintained on a 12 h light, 12 h dark cycle, at 30°C. While many bees emerged from cells completely before transfer, some bees were still inside their uncapped cell during transfer, so cages usually had some uncapped cells. Survival was noted once daily, and any dead bees were removed immediately. Each cage was provided with one modified 1.5 ml microcentrifuge tube with 30% sterile sucrose solution, and the alfalfa leafcutting bees were fed *ad libitum*, with the sucrose solution tube replaced once every 2–3 days. For UPR induction, at 7–9 days of age, bees were fed 30% sterile sucrose solution with or without tunicamycin at 30 µmol l^−1^ (Sigma, St Louis, MO, USA) as previously performed for honey bees (Adames et al., 2020; Johnston et al., 2016; Shih et al., 2021). The solute for tunicamycin is DMSO, so equivalent amounts of DMSO were added to the food of the control group in tunicamycin experiments. After 48 h, bees were cold anesthetized, and midguts were removed and placed into RNAlater (Invitrogen) for storage prior to gene expression analysis of individual bees. All alfalfa leafcutting bee tunicamycin experiments were performed for a minimum of four independent trials with similar results.

### Alfalfa leafcutting bee dye feeding assay

The dye feeding assay was developed based on a protocol for analyzing the feeding patterns of *D. melanogaster* (Edgecomb et al., 1994). At 7–9 days of age, bees were fed 30% sterile sucrose solution alone or containing 0.16% Acid Blue 9 (TCI America, Portland, OR, USA). After 48 h, bees were cold anesthetized, and midguts were removed and placed in 200 μl water. Following dissection, midguts were crushed using sterile plastic pestles (Fisher, Waltham, MA, USA). Tubes containing the crushed midguts were centrifuged at 6000 rpm for 5 min and the absorbance of the resulting supernatants was determined using a Spark 10M spectrophotometer (Tecan, Männedorf, Switzerland) at 628 nm.

### Ortholog screening of the *M. rotundata* genome

UPR pathway gene candidates from *A. mellifera* were used to find orthologs in the alfalfa leafcutting bee genome using the BLAST family of search functions (https://blast.ncbi.nlm.nih.gov/Blast.cgi).

### RNA isolation, reverse transcription and quantitative PCR for gene expression analysis

RNA was prepared from bees from the described populations by manually crushing the tissue of interest with a disposable pestle in Trizol Reagent (Invitrogen) and extracting the RNA as per the manufacturer's instructions. RNA was subsequently DNAseI treated by RQ1 RNase-Free DNase (Promega, Madison, WI, USA) and quantified. cDNA was synthesized using approximately 1 µg of RNA with the iScript cDNA Synthesis Kit (BioRad, Hercules, CA, USA). Typically, 1 µl of cDNA was then used as a template for quantitative PCR to determine the level of expression of genes of interest using the iQ SYBR Green Supermix (BioRad) in an iCycler thermal-cycler (BioRad). Primer sequences for transcripts of genes of interest are in Table S1A or from Adames et al. (2020), Johnston et al. (2016) and Shih et al. (2021). The difference between the threshold cycle number for *β-actin* and that of the gene of interest was used to calculate the level of expression of that gene relative to *β-actin* using the 2^−Δ*C*_T_^ method (Schmittgen and Livak, 2008).

### Alfalfa leafcutting bee *Xbp1* mRNA splicing

cDNA from above was used as a template for PCR using primers (forward, 5′-GCCAGAGTCGTGTGCTGTAG-3′ and reverse 5′-GCTCCTGGGGTTAGTGTCAG-3′) that spanned the predicted *Xbp1* splice sites. PCR products were run on a 2.5% agarose gel to separate spliced from unspliced *Xbp1*. DNA was purified from bands representing amplicons from unspliced and spliced forms of the *Xbp1* transcript using a Gel Extraction Kit (Qiagen, Hilden, Germany), cloned into pDrive (Qiagen) and sequenced (Genewiz, South Plainfield, NJ, USA). The honey bee splicing assay was performed as in Johnston et al. (2016).

### Statistical analysis

For statistical analysis of gene expression, GraphPad Prism was used. Data were log_10_ transformed and compared using unpaired *t*-tests with Welch's correction when values fitted normal distributions or Mann–Whitney *U* non-parametric tests when values did not fit normal distributions. Normality was assessed using Shapiro–Wilk tests. When more than two groups were being compared, data were compared using one-way ANOVA with Tukey's multiple comparison test when values fitted normal distributions (Fig. S1B) or a Kruskal–Wallis test with Dunn's multiple comparisons test when there was a non-normal distribution (Fig. S4E,F). In the statistical treatment of targeted NMR metabolomic data, IBM SPSS 28.0.1.0 (142) was used, all outliers were retained, and there were no missing values. The sarcosine concentrations of the control and halofuginone groups were compared with an independent samples *t*-test following Levene's test and the Shapiro–Wilk test. Detailed descriptions of the statistical analysis used for figures can be found in Table S1B.

## RESULTS

### *Gnmt* is upregulated by diverse proteotoxic stresses in honey bees

Members of the *l(2)efl* family of small heat shock proteins were previously identified as gene targets common to responses to diverse stressors in honey bees using transcriptome profiling (RNASeq) (Adames et al., 2020; Bach et al., 2021; Flores et al., 2021). When we analyzed these gene lists to define additional targets of a possible gene expression program shared by all stress responses, we observed *Gnmt* as a differentially expressed gene in all data RNASeq sets (Table S1C). *Gnmt* encodes a central enzyme within methionine metabolism, catalyzing the transfer of a methyl group from SAM to glycine to form sarcosine and SAH. GNMT is thought to be the principal regulator of the SAM/SAH ratio in cells in diverse organisms and therefore serves as a critical metabolic switch regulating cellular methylation reactions (Luka et al., 2009) (Fig. 1A).

To confirm these findings, we first examined heat shock-dependent induction of *Gnmt* in midgut tissue from honey bees kept at either at 35 or 45°C for 4 h and observed increased levels of *Gnmt* transcripts after heat shock in this tissue (Fig. 1B). We also confirmed that aminoacyl tRNA synthetase (AARS) inhibition (via halofuginone feeding), or ER stress (induced by tunicamycin feeding) increase *Gnmt* expression after 48 h of treatment compared with controls (Fig. 1C,D). We were interested in determining whether other forms of cellular stress also induced *Gnmt* expression. We showed that ribosome inhibition (by cycloheximide feeding; Fig. 1E) and proteasome inhibition (by ixazomib feeding; Fig. 1F) caused upregulation of *Gnmt* in midgut tissue relative to control bees receiving only sucrose solution after 48 h of treatment. Finally, we showed that induction of oxidative stress (via sodium arsenite feeding for 24 h) caused upregulation of *Gnmt* in midgut tissue relative to control bees receiving sucrose solution alone (Fig. 1G).

While orally administered treatments appear to only reliably affect the digestive tract, we can apply thermal stress to all tissues equally. Thus, we examined *Gnmt* expression in multiple tissues after heat shock, including head tissue (predominantly brain and sensory organ tissue), midgut, thorax tissue (predominantly flight muscle) and abdominal wall (predominantly fat body) tissues, after exposure to 35 or 45°C for 4 h (Fig. S1A). We found significant induction only in midgut tissue, suggesting that *Gnmt* induction may be part of a stress response that is tissue specific. We also took advantage of a previous dataset in which we had matched samples from attendant workers and reproductive queens (Shih et al., 2020) maintained at 35 or 45°C for 4 h to determine whether *Gnmt* is induced in queens. We found that *Gnmt* is increased in queen midguts to a degree comparable to that of workers following thermal stress (Fig. S1B).

**Fig. 2.**
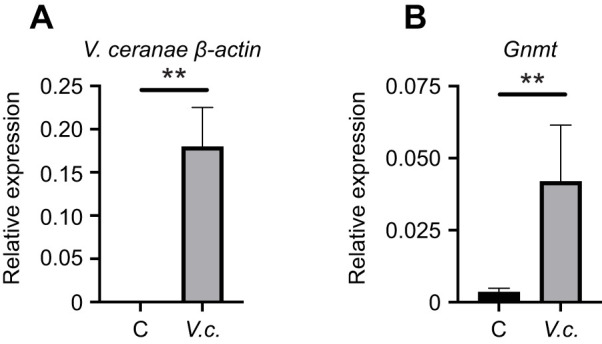
***Gnmt* is induced by *Vairimorpha ceranae* infection in honey bees.** (A) *Vairimorpha* (*Nosema*) *ceranae* infection levels (assessed by *V. ceranae β-actin* levels relative to honey bee *β-actin*) 9 days after newly eclosed bees were exposed to *V. ceranae* (*V.c.*, *n*=6) or left uninfected (control, C; *n*=6). (B) Transcript levels of *Gnmt* relative to honey bee *β-actin* in midgut tissue from control or *V. ceranae*-infected bees. Data (means± s.e.m.) represent expression of the genes of interest calculated using the 2^−Δ*C*_T_^ method for individual bees. Statistical significance is indicated by asterisks (**P<0.01).

Thus far, all of the stressors examined have been abiotic in nature. Therefore, we wanted to examine whether a biotic stressor, such as an infection, might lead to increased *Gnmt* expression. We looked at *Gnmt* expression in honey bees that had been infected with *V. ceranae*, a microsporidia species that causes individual disease in honey bees and can contribute to colony collapse (Snow, 2022). Bees were infected with *V. ceranae* 1 day post-eclosion via a recently described soak method or left uninfected (Parrella et al., 2024). On day 9 post-infection, we harvested midgut tissue and first measured levels of a *V. ceranae* transcript to confirm robust infection by this parasite (Fig. 2A). We then measured *Gnmt* transcript levels in these samples and found that expression of this gene was increased in infected bees when compared with uninfected bees (Fig. 2B).

### Changes in metabolome correlate with transcriptional changes in methylation cycle

**Fig. 3.**
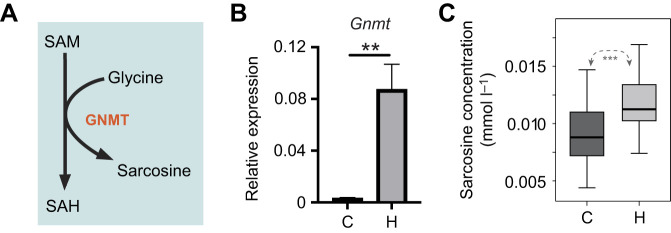
**Halofuginone induces integrated stress response (ISR) target genes and impacts survival in newly eclosed honey bees.** (A) Schematic diagram of the methionine cycle reaction catalyzed by GNMT. (B) Transcript levels of *Gnmt* relative to *β-actin* in midgut tissue from newly eclosed bees feeding on sucrose solution containing halofuginone (H; *n*=8) or DMSO vehicle (control, C; *n*=8) for 4 days. Data (means±s.e.m.) represent expression of the genes of interest calculated using the 2^−Δ*C*_T_^ method for individual bees. (C) A box and whisker plot (median, first and third quartiles, no outliers, whiskers denote minimum/maximum) of midgut sarcosine concentration (relative to a 350 μl volume) for control and halofuginone-treated bees. Statistical significance is indicated by asterisks (**P<0.01, ***P<0.001).

Increased *Gnmt* mRNA levels would be expected to impact levels of reactants and products in the reaction it catalyzes. Sarcosine (along with SAH) is a product of the reaction catalyzed by GNMT in which glycine is methylated using SAM as a methyl donor (Fig. 3A). We first confirmed that the effects of halofuginone on *Gnmt* gene expression in newly eclosed honey bees were similar to those observed above in older bees (Fig. 3B). We then used a NMR strategy for sampling the aqueous metabolome in either whole bees or bee organs, which has been pursued previously (McDevitt et al., 2021; Pizzorno et al., 2021), to examine the midgut levels of metabolites in response to halofuginone feeding. We found that the amino acid sarcosine was increased in the midgut (*P*<0.001, Fig. 3C). Full results and analysis of these data are forthcoming in another paper (N.C.R., J.R.-C., H.V.K., R. M. Cho, N. L. Lovinger, F. Wague, A. J. Loptakin, N. G. Texeira, M.E.F., D.R. and J.W.S., unpublished).

### UPR pathway components are conserved in the alfalfa leafcutting bee

**Fig. 4.**
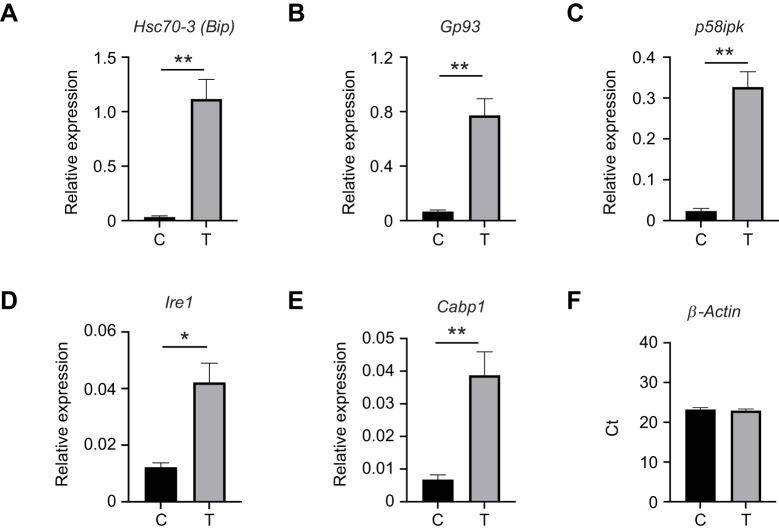
**Alfalfa leafcutting bee unfolded protein response (UPR) target genes are induced by tunicamycin.** (A–E) Transcript levels of UPR target genes representing chaperones *Hsc70-3* (A), *Gp93* (B), *p58ipk* (C), UPR pathway component *Ire1* (*D*) and the disulfide bond-generating enzyme *Cabp1* (E) relative to *β-actin* in midgut tissue from individual control (DMSO vehicle, C; *n*=5) or 30 μmol l^−1^ tunicamycin-treated bees (T; *n*=5) after 48 h. The difference between the threshold cycle number for *β-actin* and that of the gene of interest was used to calculate the expression level of that gene relative to *β-actin* using the 2^−Δ*C*_T_^ method. (F) Threshold cycle number for *β-actin* for these bees. Data are means±s.e.m. Statistical significance is indicated by asterisks (*P<0.05, **P<0.01).

To determine whether the novel stress-responsive gene *Gnmt* behaves similarly in other bee species, we first characterized one of the model cellular responses used in honey bees in lab-reared adults of the solitary alfalfa leafcutting bee (*Megachile rotundata*) in comparison with age-matched honey bees. We chose the cellular response to ER stress triggering the UPR because of the high conservation of this pathway. Analysis of the *M. rotundata* genome revealed that the core signaling components of the IRE1, PERK and ATF6 pathways are conserved (Table S1D). We tested a number of putative UPR target genes based on previous results from the honey bee (Adames et al., 2020; Johnston et al., 2016). We first confirmed that alfalfa leafcutting bees had robust survival in our caging system and found ∼80% survival of eclosed adults for 9 days, with most death occurring early post-eclosion (Fig. S2A). We then confirmed that bees were consuming sucrose solution at 7 days post-eclosion using Acid Blue 9 dye (Fig. S2B). Once conditions were established, we exposed cages of bees at 7 days post-eclosion to either sucrose solution containing DMSO vehicle control or sucrose solution containing 30 µmol l^−1^ tunicamycin. Many of the putative target genes we studied before (Adames et al., 2020; Johnston et al., 2016) [including genes from each of the following groups: chaperones (*Hsc70-3*, *Gp93*, *p58ipk*), UPR pathway components (*Ire1*) and disulfide bond generating enzymes (*CaBP*)] were upregulated significantly in the alfalfa leafcutting bee digestive tract after 48 h of 30 µmol l^−1^ tunicamycin treatment relative to bees consuming sucrose solution alone (Fig. 4A–E). We chose *β-actin* as a reference gene (Xu et al., 2021) and found that its levels were not altered by treatment (Fig. 4F). Two additional reference genes (*Rps18* and *Gapdh*) were tested and their expression did not differ significantly by treatment (tunicamycin treated versus untreated, Fig. S3C,D) and similar patterns of induced expression were found when these other reference genes were used for normalization (data not shown).

Additionally, we tested similarly aged honey bees (starting 7 days post-eclosion) for induction of these target genes and found that chaperones (*Hsc70-3*, *p58ipk*, *Gp93*), UPR pathway components (*Ire1*) and disulfide bond generating enzymes (*CaBP*) were upregulated significantly in the honey bee digestive tract after 48 h of 30 µmol l^−1^ tunicamycin treatment relative to bees consuming sucrose solution alone (Fig. S3A–F) as reported previously for older bees (Adames et al., 2020; Johnston et al., 2016).

### *Xbp1* mRNA splicing occurs in response to tunicamycin treatment

**Fig. 5.**
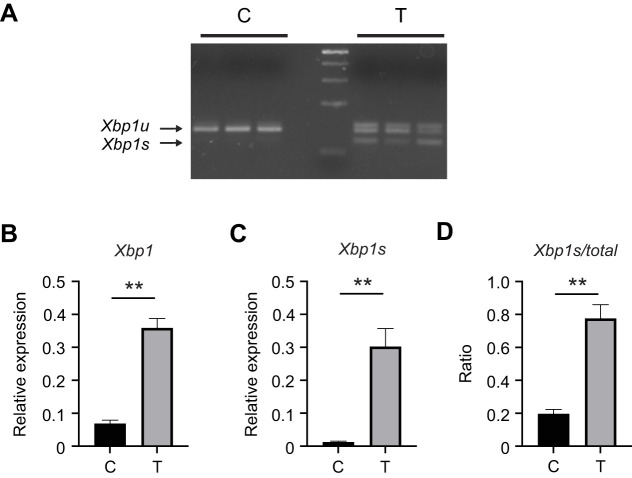
***Xbp1* mRNA splicing in alfalfa leafcutting bees is induced by tunicamycin.** (A) *Xbp1* mRNA splicing by IRE1 in midgut tissue from individual bees fed sucrose solution with (T; *n*=5) or without (DMSO vehicle control, C; *n*=5) 30 μmol l^−1^ tunicamycin for 48 h. *Xbp1u*, unspliced *Xbp1*; *Xbp1s*, spliced *Xbp1*. (B,C) Transcript levels of total *Xbp1* (B) or spliced *Xbp1* alone (C) relative to *β-actin* in midgut tissue from individual control (*n*=5) and 30 μmol l^−1^ tunicamycin-treated bees (*n*=5) after 48 h. (D) Ratio of spliced *Xbp1* to total *Xbp1* in midgut tissue from individual control (*n*=5) and 30 μmol l^−1^ tunicamycin-treated bees (*n*=5) after 48 h. The difference between the threshold cycle number for *β-actin* and that of the gene of interest was used to calculate the expression level of that gene relative to *β-actin* using the using the 2^−Δ*C*_T_^ method. Data are means± s.e.m. Statistical significance is indicated by asterisks (**P<0.01).

*Xbp1* mRNA splicing is a critical step that is often monitored for evidence of IRE1 pathway activation. Using the non-canonical intron structures predicted for the alfalfa leafcutting bee *Xbp1* mRNA (Johnston et al., 2016) to design primers spanning the predicted intron, we were able to differentiate unspliced and spliced *Xbp1* mRNA via PCR and gel analysis (Fig. 5A). Alfalfa leafcutting bees fed 30 µmol l^−1^ tunicamycin for 48 h had two abundant PCR products of ∼116 and ∼93 base pairs (bp), representing unspliced (*Xbp1u*) and spliced (*Xbp1s*) mRNA species, respectively, while bees fed sucrose solution containing vehicle had a single dominant PCR product of ∼116 bp at 48 h (band identities confirmed by purification and sequencing). In addition to splicing after tunicamycin treatment, total *Xbp1* mRNA was upregulated significantly in the alfalfa leafcutting bee digestive tract after 48 h of tunicamycin treatment relative to bees consuming sugar syrup alone (Fig. 5B). To examine splicing more quantitatively, we designed a primer set that amplifies only the spliced version of the *Xbp1* transcript (described in more detail for honey bees below). Using this primer set and the primer set that amplifies all *Xbp1*, we observed that while total *Xbp1* transcript increased modestly after tunicamycin treatment, the ratio of spliced to total *Xbp1* increased significantly after tunicamycin treatment (Fig. 5B–D).

We again tested similarly aged honey bees for *Xbp1* splicing using conventional PCR and observed *Xbp1* mRNA splicing after tunicamycin treatment (Fig. S4A) in accordance with our previous findings in older bees (Adames et al., 2020; Johnston et al., 2016). We then tested the honey bees that were 7 days post-eclosion using the *Xbp1s* primer set and the primer set that amplifies total *Xbp1* and observed that while total *Xbp1* transcript increased modestly after tunicamycin treatment, the ratio of spliced to total increased substantially after tunicamycin treatment (Fig. S4B–D). Using the primer set that amplifies only the spliced version of the *Xbp1* transcript, we observed that the amount of *Xbp1s* increased dramatically in a dose-dependent manner after tunicamycin treatment in a way similar to the amount of the canonical target gene *Hsc70-3* (Fig. S4E,F).

### UPR pathway induces select *l(2)efl* genes in the alfalfa leafcutting bee

**Fig. 6.**
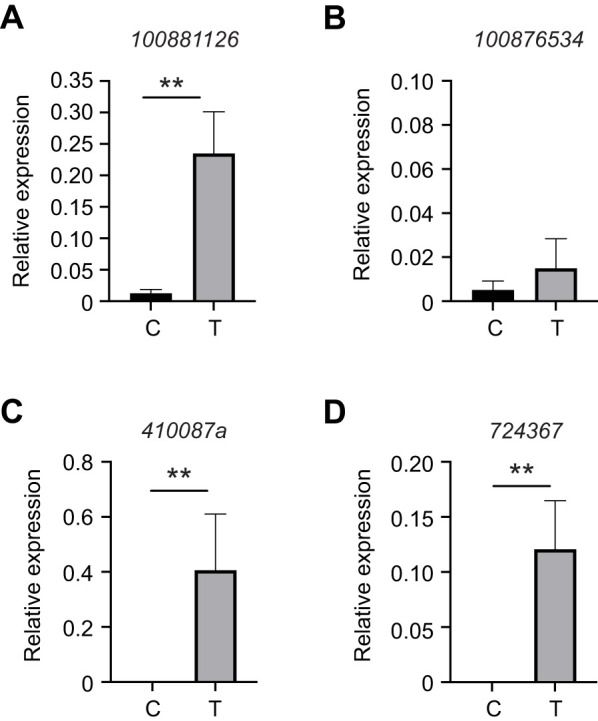
**Select alfalfa leafcutting bee and honey bee *l(2)efl* genes are induced by tunicamycin.** (A,B) Transcript levels of the *l(2)efl* genes 100881126 (A) and 100876534 (B) relative to *β-actin* in midgut tissue from individual control (DMSO vehicle, C; *n*=5) or 30 μmol l^−1^ tunicamycin-treated (T; *n*=5) alfalfa leafcutting bees after 48 h. (C,D) Transcript levels of the *l(2)efl* genes *410087a* (C) and *724367* (D) relative to *β-actin* in midgut tissue from adult honey bees captured at the landing board and fed sucrose solution with (T; *n*=10) or without (DMSO vehicle control, C; *n*=10) 30 μmol l^−1^ tunicamycin for 48 h. Data (means±s.e.m.) represent expression values of the genes of interest calculated using the 2^−Δ*C*_T_^ method for individual bees. Statistical significance is indicated by asterisks (**P<0.01).

We previously found that members of the *l(2)efl* family of genes are induced by diverse stressors in honey bees (Shih et al., 2021). We showed that two of these (*410087a* and *724367*) could be useful as stress biomarker genes in honey bees responding to diverse types of stress (Cho et al., 2022; Shih et al., 2021). Additionally, we found that *M. rotundata* and *Osmia lignaria*, which are both in the Megachilidae family, share *shsp* gene number and structure with *A. mellifera* (Shih et al., 2021). We tested whether UPR activation induced the transcription of the homologs of two of the *l(2)efl* genes shown to be UPR targets in honey bees. We found that only one of the *l(2)efl* genes (*100881126* corresponding to *410087a* in the honey bee) was upregulated significantly in the alfalfa leafcutting bee digestive tract after 48 h of 30 µmol l^−1^ tunicamycin treatment relative to bees consuming sucrose solution alone (Fig. 6A,B). Expression of the *l(2)efl* gene *100876534* (corresponding to *724367* in the honey bee) was not increased by tunicamycin treatment. Interestingly, when we tested similarly aged honey bees, we found both of the tested *l(2)efl* genes (*410087a* and *724367*) were upregulated significantly in the honey bee digestive tract after 48 h of 30 µmol l^−1^ tunicamycin treatment relative to bees consuming sucrose solution alone (Fig. 6C,D).

### ER stress activates the *Gnmt* gene in alfalfa leafcutting bees

**Fig. 7.**
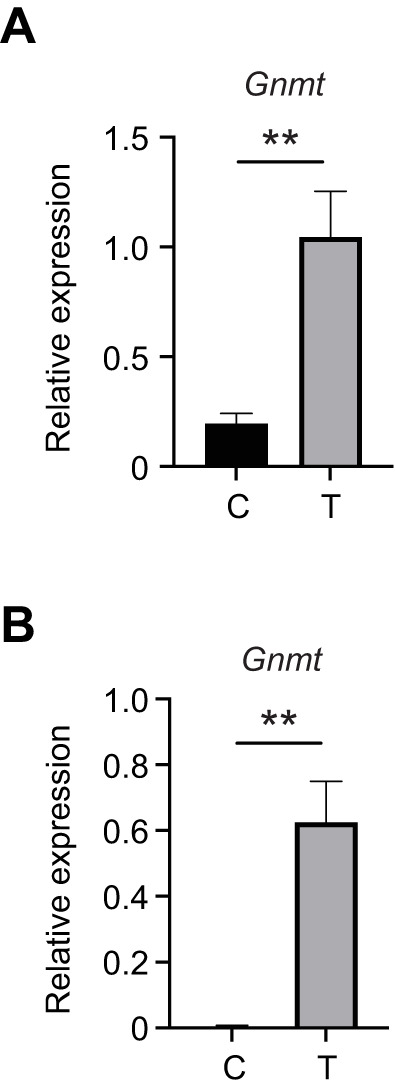
**Alfalfa leafcutting bee and honey bee *Gnmt* is induced by tunicamycin.** Transcript levels of *Gnmt* relative to *β-actin* in midgut tissue from individual control (DMSO vehicle, C) or 30 μmol l^−1^ tunicamycin (T)-treated alfalfa leafcutting bees (*n*=5 control, *n*=5 tunicamycin; A) and honey bees (*n*=10 control, *n*=10 tunicamycin; B) after 48 h. The difference between the threshold cycle number for *β-actin* and that of the gene of interest was used to calculate the expression level of that gene relative to *β-actin* using the 2^−Δ*C*_T_^ method. Data are means±s.e.m. Statistical significance is indicated by asterisks (**P<0.01).

To determine whether *Gnmt* was upregulated in response to stress in the midgut of the distantly related and solitary alfalfa leafcutting bee species, we measured the expression of this gene after tunicamycin treatment of females 7 days post-eclosion. We found that expression of *Gnmt* was increased in response to tunicamycin treatment in alfalfa leafcutting bees (Fig. 7A). To confirm that these genes can be induced in honey bees following this protocol, we also examined midguts of age-matched honey bees (7 days post-eclosion) after 30 μmol l^−1^ tunicamycin treatment for 48 h in age-matched honey bees and observed induction of *Gnmt* in response to tunicamycin in these bees as well (Fig. 7B).

### *Gnmt* expression may be regulated by FOXO and ATF4 in response to stress

Based on the diversity of stresses able to promote gene induction in honey bees, we were interested in exploring the potential trans factors and cis elements responsible for regulating increased gene expression. In both flies and mice, *Gnmt* is increased after a reduction in IIS signaling (Tain et al., 2020), likely mediated by the forkhead box (FOXO) transcription factor. We observed a number of consensus sites for FOXO binding (TKTTYACY; Bai et al., 2013) near the *Gnmt* transcriptional start site (TSS) in both honey bees and alfalfa leafcutting bees (Fig. S5).

FOXO is the key transcription factor involved in the IIS pathway responding to both nutrient levels and stress (Semaniuk et al., 2021a,b). We also looked for binding sites for other transcription factors associated with the stress pathways we found to induce *Gnmt* expression. We found one putative ATF4-binding CREB response element binding site (defined as either TGACGT or TTKCATCAK) downstream of the *Gnmt* TSS in both species. The transcriptional regulator controlling the oxidative stress response, NRF2 (and its homologs), is responsible for promoting resistance to oxidative stress in *C. elegans*, *D. melanogaster* and mammals (reviewed in Sykiotis and Bohmann, 2008). NRF2-responsive genes possess the consensus binding site TGAYNNNGC, which makes up the core of the antioxidant responsive element (ARE). We found evidence of a single ARE in the honey bee and alfalfa leafcutting bee *Gnmt* promoters and an additional ARE site downstream of the TSS in alfalfa leafcutting bees (Fig. S5). When examining the mRNA for regulatory features, we found that the predicted transcripts of both the honey bee and alfalfa leafcutting bee *Gnmt* genes possess significant 5′ and 3′ UTR sequences with the 5′ UTR possessing an upstream open reading frame (uORF; Fig. S5).

## DISCUSSION

### GNMT as a stress-inducible metabolic switch regulating cellular methylation

Here, we report the identification of *Gnmt* as a novel stress-inducible gene upregulated by diverse stressors using unbiased approaches in honey bees. We found that *Gnmt* expression is induced by both abiotic and biotic stressors. In bees that were infected with *V. ceranae*, a microsporidia species that causes individual disease in honey bees and can contribute to colony collapse (Snow, 2022), *Gnmt* expression was increased after 9 days of infection when compared with similarly aged bees that were not infected. *Vairimorpha ceranae* is documented to cause many changes to infected epithelial cells at the biochemical and cell biological level (reviewed in Snow, 2022) and defining whether infection-induced induction of *Gnmt* is beneficial or detrimental in response to infection will be important. sHSPs represent another class of potential biomarkers identified by our lab using this same approach (Shih et al., 2021) that have now been shown by multiple groups to be induced by abiotic stress (Cho et al., 2022; Goblirsch and Adamczyk, 2022; McAfee et al., 2020a,b; Parrella et al., 2024; Shih et al., 2021) and biotic stress as they comprise part of the antiviral response in multiple bee species (Brutscher et al., 2017; McMenamin et al., 2018). We extend this finding to show that *Gnmt* expression is also triggered by stress in alfalfa leafcutting bees. GNMT plays a key role in the methionine cycle (Sanderson et al., 2019), in which it is thought to be the main regulatory switch controlling the SAM/SAH ratio. Evidence for the key role of GNMT in this cycle is found in diverse organisms. In humans, deficiency in the *Gnmt* gene causes hypermethioninemia with increased levels of SAM (Mudd et al., 2001). Mice lacking *Gnmt* have elevated methionine levels, a dramatically increased SAM to SAH ratio, and multiple other metabolic abnormalities (Hughey et al., 2018; Luka et al., 2006). Fruit flies deficient in *Gnmt* also have an increased SAM/SAH ratio (Obata and Miura, 2015; Obata et al., 2014; Tain et al., 2020) and increased sarcosine is one of the most pronounced observed metabolic changes (Obata and Miura, 2015; Obata et al., 2014). In accordance with the results from *Drosophila*, we also found an increase in sarcosine levels. These results show that by at least one metric, our observation of stress-induced *Gnmt* expression results in the expected biochemical changes*.*

The current thought is that effects of methionine and its cycle may primarily impact transmethylation, which refers to the myriad of methylation reactions performed by diverse enzymes using SAM as a methyl donor. In fact, most methylation in cells takes place by SAM*-*dependent methyltransferases. These methyltransferases catalyze the often reversible methylation reactions of DNA, RNA, proteins and small molecules (Abdelraheem et al., 2022). Some key examples include the methylation of DNA which directs epigenetic regulation of gene expression, the methylation of RNA which regulates many of the processes involved in RNA handling (e.g. splicing and protein synthesis), the methylation of proteins to alter their function (e.g. histone methylation to regulate gene expression) and the methylation of diverse small molecules in the service of metabolism or to alter a molecule's chemical properties. Methylation of RNA molecules is one type of methylation reaction that is highly conserved, SAM dependent and involved in regulating many aspects of RNA biology (Zhang et al., 2021). Interestingly, the N^6^-methyladenosine (m^6^A) methylation of RNA has been implicated cellular stress in many contexts including cellular stress (Wilkinson et al., 2021) and has recently been shown to be involved in honey bee biology (Wang et al., 2021). Specifically, differential m^6^A methylation was shown to post-transcriptionally regulate gene expression in honey bee larval development and caste differentiation (Wang et al., 2021). Further work will be necessary to disentangle potential changes in the methylation of the molecules above resulting from stress-induced increases in *Gnmt* expression and activity in bees.

GNMT’s role in the regulation of methionine pathway metabolism is thought to be significant in terms of governing these SAM-dependent transmethylation reactions. By using SAM as the methyl donor for conversion of glycine to sarcosine, Gnmt activity reduces SAM levels in favor of SAH. As SAM is the primary methyl donor for a whole host of cellular methylation reactions (Parkhitko et al., 2020), a concomitant reduction in transmethylation would be expected after *Gnmt* induction. Although changes in cellular methylation reactions are likely a primary consequence of *Gnmt* upregulation and enzymatic conversion of SAM and glycine to sarcosine and SAH, it is important to note that the methionine cycle (Sanderson et al., 2019) is directly linked to a number of other metabolic processes which might also be affected. These pathways include the transsulfuration pathway/gamma-glutamyl cycle (Bonifácio et al., 2021), the one carbon metabolism/folate cycle (Ducker and Rabinowitz, 2017) and polyamine biosynthesis (Casero et al., 2018). *Gnmt* overexpression may play key roles in the regulation of these aspects of cell biology as well, and future work will be required to define the consequences of increased *Gnmt* expression on cell biology in bees.

In the fly, GNMT-mediated regulation of SAM levels is driven by genetic lesions that induce tissue damage (Kashio et al., 2016; Obata et al., 2014). Dietary methionine acting via SAM levels has also been implicated in regulating protein synthesis and tissue regeneration in the fly (Obata et al., 2018). Prior to our study, *Gnmt* upregulation had not previously been associated with cellular stress responses. It is likely that damage occurring via genetic means or caused by the triggers of cellular stress responses used here (such as thermal stress, ER stress, etc.) could result in the activation of a non-specific stress pathway with shared target genes such as *Gnmt*. One likely candidate is the IIS pathway. Multiple lines of evidence suggest that *Gnmt* levels are controlled by insulin in flies (Obata and Miura, 2015; Obata et al., 2014; Tain et al., 2020) and the IIS pathway may be activated during these model stress responses in bees. This link with the IIS pathway fits with other data showing that *Gnmt* expression increases with age and that its overexpression increases lifespan (in flies: Obata and Miura, 2015; Tain et al., 2020). Other genetic and chemical perturbations have also implicated the methionine pathway as being a critical mediator of lifespan extension (Parkhitko et al., 2019, 2020). For example, *S*-adenosyl-l-homocysteine hydrolase (SAHH), which converts SAH to adenosine and homocysteine, as mediated by inactivation of its negative regulator, also results in increased lifespan (Parkhitko et al., 2016). In the fly, the fat body is thought to be the primary organ for GNMT-mediated control of systemic SAM levels (Obata and Miura, 2015; Obata et al., 2014; Tain et al., 2020). Similarly, in mammals, Gnmt regulates physiological SAM levels through action in the liver (Luka et al., 2009), which is often considered functionally analogous to the insect fat body. We observed increased *Gnmt* expression and metabolite differences in the digestive tract. While there are many potential reasons for this difference, the most likely appears to be that the perturbations used in our system cause a tissue-specific response in the midgut rather than a systemic alteration of SAM levels. Supporting this idea, most of our perturbations were localized to the midgut via drug delivery. SAM levels have already been shown to have a special role in regulating stem cell proliferation in the fly midgut (Obata et al., 2018). Although this effect appeared not to be dependent on GNMT, it might be expected that proteotoxic stressors shown here might influence SAM levels via a different mechanism from the DNA damage perturbation used (Obata et al., 2018). To interpret these differences, the use of experiments designed to allow for more direct comparison of the two models is warranted.

### Shared stress responses in diverse bee species

Our study supports the idea that there is considerable overlap in the stress responses of disparate bee species. Furthermore, our results confirm that honey bees can provide a useful model for learning about the mechanism of stress resistance in other bee species. In addition to finding *Gnmt* as a stress-induced target gene in both bee species, we also observed significant conservation in the function of the canonical UPR in the two different bee species with disparate life histories. Based on the high conservation of UPR architecture and apparent function in honey bees as compared with other insects (Adames et al., 2020; Johnston et al., 2016), it is to be expected that such a conserved pathway would be quite similar in another bee species. The alfalfa leafcutting bee possesses the same unusual sequence at the 5′ end of the *Xbp1* mRNA non-canonical intron stem-loop recognized by IRE1 as other bee species (Johnston et al., 2016). Despite the validation of honey bees as a model for other bees shown here, previously published work suggests that studies using honey bees cannot take the place of research on other bee species (Wood et al., 2020), and our study provides some support for this idea as well. Specifically, we observed differences in UPR induction of select *l(2)efl* genes between the two species. The expression levels of these sHSP (at both the transcript and protein levels) represent candidate biomarkers of stress in honey bees (Cho et al., 2022; McAfee et al., 2020a,b; Shih et al., 2021). With some exceptions, our previous phylogenetic analysis of the sHSP proteins in other bee species shows a high degree of conservation of this family among bees with different life histories. Critical for the current work, we observed that *M. rotundata* and *O. lignaria*, which are both in the Megachilidae family, share *shsp* gene number and structure with *A. mellifera* (Shih et al., 2021). Thus, the expectation would be that these genes would be induced by stressors similarly in these two bee species. However, contrary to our expectations, we found that only one of the two examined *l(2)efl* genes was induced in alfalfa leafcutting bees despite both being strongly induced in honey bees of the same age. It will be of interest to explore potential causes and consequences of such differential responses. While only relatively subtle differences were found using the candidate gene approach described here, it seems reasonable to speculate that a more unbiased approach, such as RNASeq, might yield more differences of obvious biological significance in the stress responses of these two species. However, more importantly, these data suggest that there are real differences in cellular responses between bee species. While honey bees can provide important insight into overall bee biology, studies on diverse bee species (Wood et al., 2020) will be required to fully understand global declines in bee populations (Zattara and Aizen, 2021).

In addition to the importance of understanding how honey bees are affected by and respond to various stressors, there is great interest in understanding the factors that influence the health of non-*Apis* bee species (López-Uribe et al., 2019). This is especially true for solitary bee species, which account for over 90% of all bee species (Danforth et al., 2019). Our previous work supported the findings of other groups that expression of *l(2)efl* genes could represent ideal biomarkers of stress in honey bees (McAfee et al., 2020a,b; Shih et al., 2021). This study suggests that one of these genes (*100881126*, homologous to honey bee *410087 V. ceranae*) is induced at a very high level in response to at least one stress. Thus, while not currently validated as a valuable indicator of exposure to stressors naturally encountered by alfalfa leafcutting bees (or honey bees), our results suggest that sHSP may provide potential biomarkers of stress in some non-*Apis* bee species as well as honey bees.

Native to Eurasia, alfalfa leafcutting bees were originally imported into the USA for alfalfa crop pollination. Alfalfa pollination by alfalfa leafcutting bees is critical for alfalfa seed production in the 4.6 billion USD per year alfalfa hay industry (Pitts-Singer and Cane, 2011). Alfalfa leafcutting bees are also important pollinators in non-agricultural ecosystems and have been facing high levels of mortality due to factors of diverse origin (Potts et al., 2016). In the USA in particular, alfalfa leafcutting bee production is so depressed that a substantial proportion of bees required for pollination must be imported each year (James and Pitts-Singer, 2013). These stressors, all of which are implicated in the loss of other bee species, include nutritional stress due to loss of appropriate forage, chemical poisoning from pesticides, changes to normal living conditions brought about through intensive management practices, and infection by arthropod parasites and pathogenic microbes (Goulson et al., 2015). Yet, despite being the second most intensively managed pollinator in the USA, our understanding of cellular stress in this bee species is incomplete. Thus, establishment of experimental systems to allow for empirical studies of diverse bee species is highly desirable. Prior experiments in alfalfa leafcutting bees have largely focused on the impacts of stressors on larval or pupal development (e.g. Cambron et al., 2021; Cambron-Kopco et al., 2022; Melicher et al., 2019; Torson et al., 2017, 2019; Yocum et al., 2018) and very few studies have been undertaken to perform experiments on adult bees using either lab-reared (Bennett et al., 2013, 2015; Cervantes and López-Martínez, 2022; Hayes and López-Martínez, 2021; James et al., 2012) or wild-captured bees (Hamblin et al., 2017). Similarly, while important studies examining stress responses at the molecular level in alfalfa leafcutting bees have been performed (Xu and James, 2012; Yocum et al., 2005, 2018), work to understand stress at the cell and molecular level in adult alfalfa leafcutting bees is still in its nascent stages, in part because of the challenges of working with these and other solitary bee species. By characterizing the UPR in alfalfa leafcutting bees in comparison with the UPR in honey bees, we advance the alfalfa leafcutting bee species as a model for cellular stress responses in solitary bees and establish a system to explore differences in cellular stress responses between bee species to provide insight into the mechanisms of bee health declines.

### Conclusions

In conclusion, we show that a key signal transduction event and a number of gene targets of the UPR are robustly induced upon ER stress in alfalfa leafcutting bees. We also provide evidence that honey bees can represent a model for better understanding cellular stress in alfalfa leafcutting bees. Discovery of *Gnmt* as a stress-activated target gene in honey bees accurately predicts it as a stress-inducible gene in the solitary bee species. The consequences of *Gnmt* induction by stress in these two bee species is likely related to its role as a key metabolic switch controlling cellular methylation reactions. Further work will be necessary to fully explore the potential changes in cellular methylation induced by stress in bees and to define whether these are beneficial or detrimental in the face of abiotic and biotic stressors. This study also suggests that the honey bee can serve as an important model for bee biology more broadly, although studies on diverse bee species will be required to understand global declines in bee populations. In particular, additional studies using more unbiased approaches with a greater number and diversity of bee species will be needed to fully understand the similarities and differences in the stress responses of different bee species.

## Supplementary Material

10.1242/jexbio.246894_sup1 Supplementary information

Table S1. Primer sequences, statistical analysis, gene list of components of the methionine cycle in *Apis mellifera*, and genes involved in the UPR in *Megachile rotundata* and *Apis mellifera*
