# Supplementary material for: Transcriptional control of a metabolic switch regulating cellular methylation reactions is part of a common response to stress in divergent bee species
Source: J Exp Biol. 2024 Jun 10;227(11):jeb246894. doi: 10.1242/jeb.246894 (PMC11415054; doi:10.1242/jeb.246894)
Supplement: Supplementary information [file jexbio-227-246894-s1.pdf]

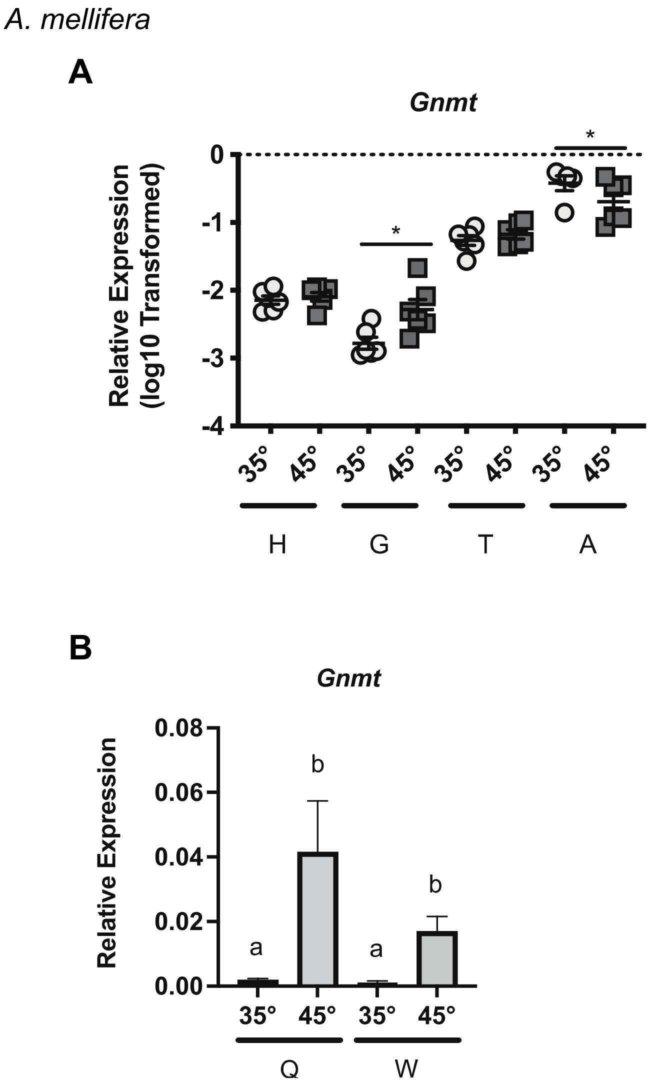

Fig. S1. Transcript levels of *Gnmt* relative to *β-actin* in head tissue (predominantly brain and sensory organ tissue), midgut, thorax tissue (predominantly flight muscle), and abdominal wall (predominantly fat body) from adult bees captured at the landing board and maintained for four hours in cages at either 35 or 45 °C (A). Symbols represent expression values of the genes of interest calculated using the $\Delta\Delta C_T$ method for individual bees. Mean ± SEM is also shown. Transcript levels of *Gnmt* relative to *β-actin* in midgut tissue from queen bees or sterile attendant worker bees maintained for four hours in cages at either 35 or 45 °C (B). Mean ± SEM is shown and represents expression values of the genes of interest calculated using the $\Delta\Delta C_T$ method for individual bees. Statistical significance is noted as $*p < 0.05$, and $**p < 0.01$ or $a \neq b$ where $p<0.05$.

Journal of Experimental Biology • Supplementary information

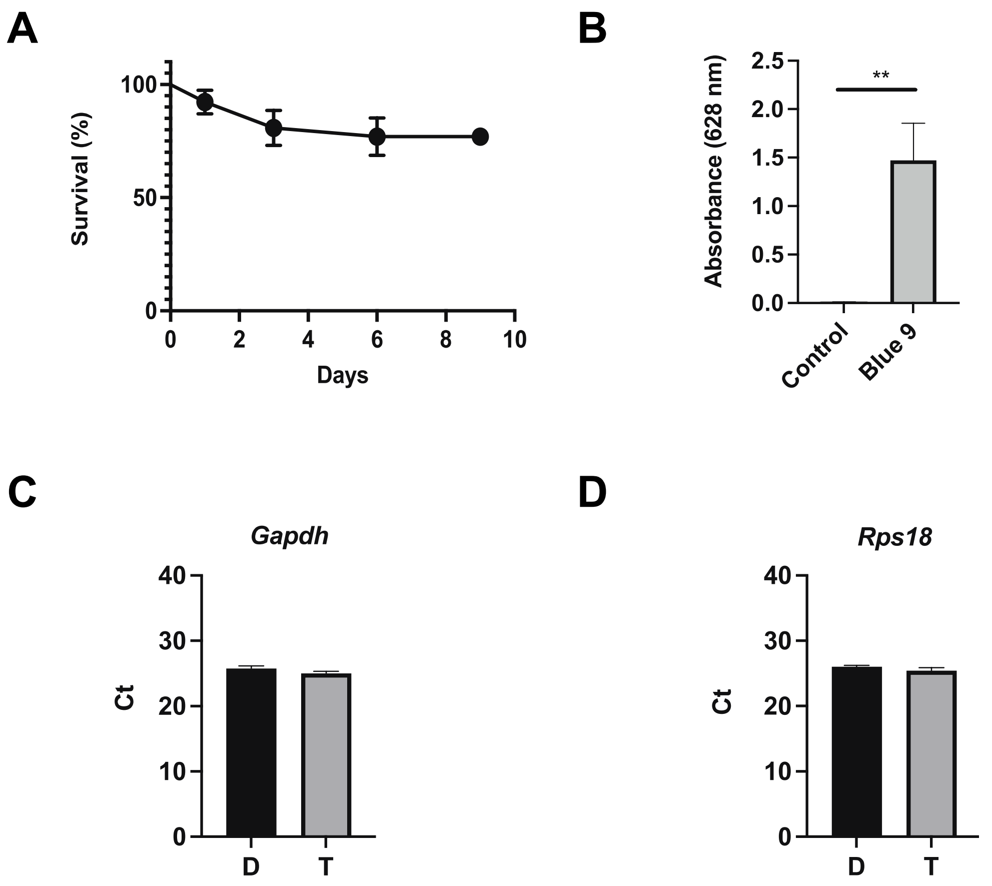

Fig. S2. Survival of individual alfalfa leafcutting bees fed sucrose solution (n=26) (A). Food consumption by individual alfalfa leafcutting bees as determined by absorbance at 628 nm in crushed midguts from bees fed 30% sucrose solution alone or containing 0.16% Acid Blue 9 for 48 hours (B). Threshold cycle number for *Gapdh* (C) and *Rps18* (D) from alfalfa leafcutting bees from experiments with tunicamycin.

Journal of Experimental Biology • Supplementary information

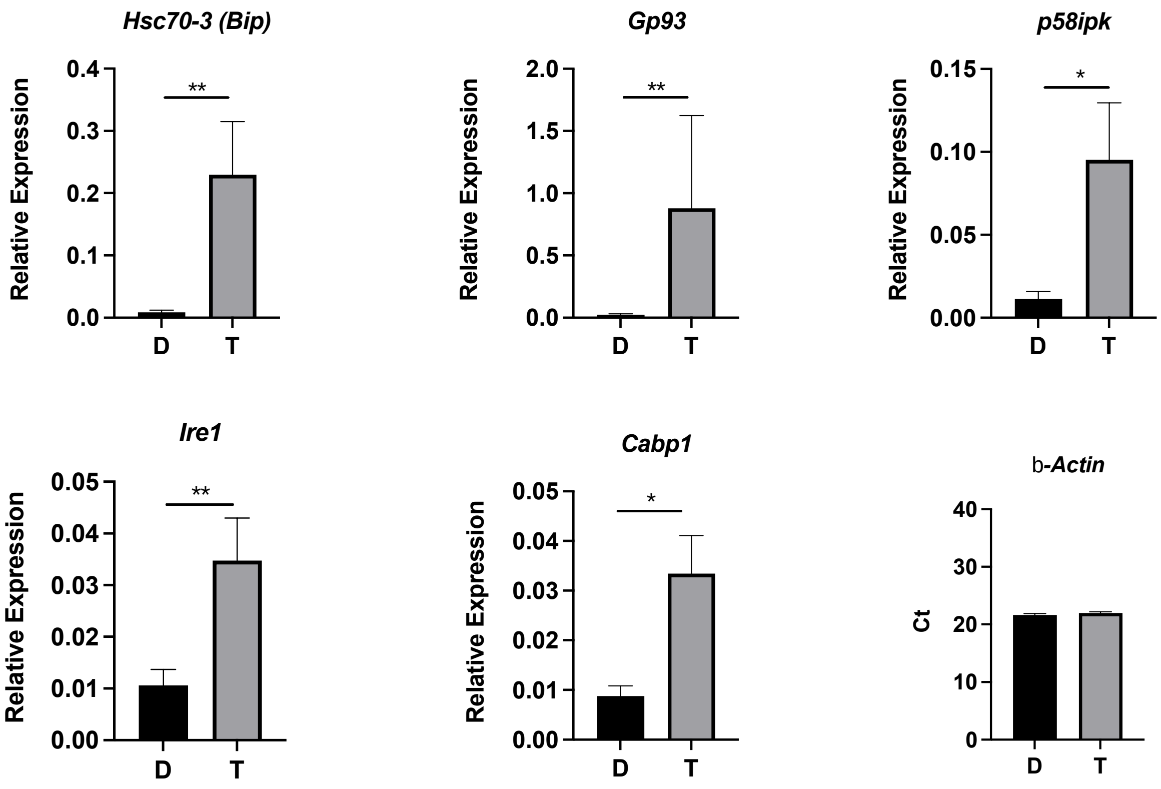

Fig. S3. Transcript levels of UPR target genes representing chaperones *Hsc70-3* (A), *Gp93* (B), *p58ipk* (C), UPR pathway component *Ire1* (*D*), and the disulfide bond generating enzyme *CaBP* (E) relative to *β-actin* in midgut tissue from individual bees with (T, n=10) or without (U, n=10) 30 μM tunicamycin treatment after 48 hours. The difference between the threshold cycle number for *β-actin* and that of the gene of interest was used to calculate the level of that gene relative to *β-actin* using the $2^{(-\Delta CT)}$ method. Threshold cycle number for *β-actin* (F) from honey bees from experiments with tunicamycin. Data is represented as Mean ± SEM. Statistical significance is noted as *p < 0.05, and **p < 0.01.

Journal of Experimental Biology • Supplementary information

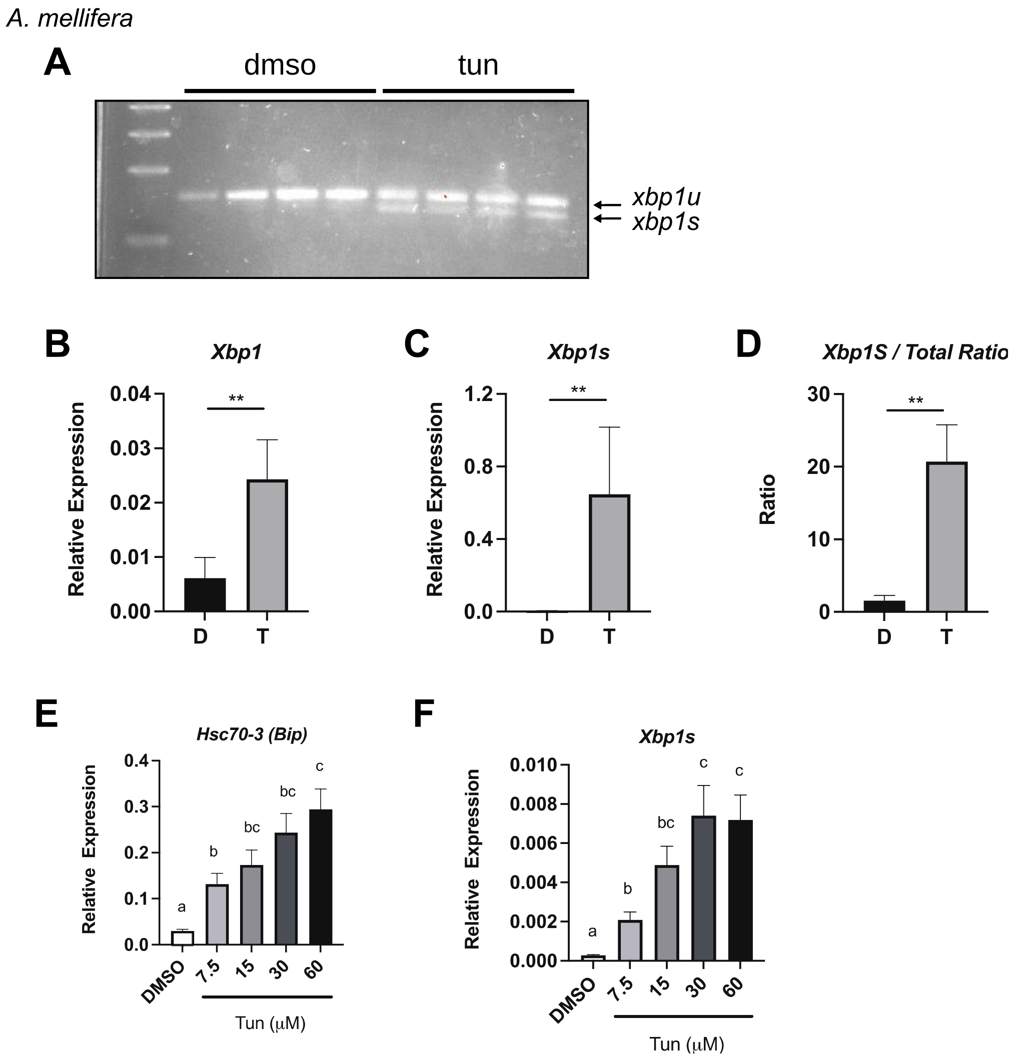

Fig. S4. *Xbp1* mRNA splicing by IRE1 in midgut tissue from individual bees fed sucrose solution with or without 30 µM tunicamycin treatment for 48 hours (A). Transcript levels of total *Xbp1* (B) or spliced *Xbp1* alone (C) relative to *β-actin* in midgut tissue from individual bees with (T, n=9) or without (D, n=10) 30 µM tunicamycin treatment after 48 hours. Ratio of spliced *Xbp1* to total *Xbp1* in midgut tissue from individual bees with or without 30 µM tunicamycin treatment after 48 hours (D). Dose responsiveness of *Hsc70-3* (E) and spliced *Xbp1* alone (F) relative to *β-actin* in midgut tissue from individual bees fed the indicated concentrations of tunicamycin for 48 hours. The difference between the threshold cycle number for *β-actin* and that of the gene of interest was used to calculate the level of that gene relative to *β-actin* using the using the $2^{(-\Delta CT)}$ method. Data is represented as Mean ± SEM. Statistical significance is noted as $*p < 0.05$, and $**p < 0.01$.

Journal of Experimental Biology • Supplementary information

(A)

HSE (Heat Shock Element) consensus sequence = GAANNTTCNNGAA
FOXO binding = TKTTYACY
UPRE (Unfolded Protein Response Element = TGACGTGR
ATF4-binding motif = TTKCATCAK, TGACGT
AREs (for antioxidant response element) = TGAYNNNGC

A. mellifera

Gnmt1 gene

ATTTATTGTATTTTTCATGAGTAAAAAAAAAAAAGTGAAAGAAAAAATATATGCTTATGCTATATCTTTTATTCAAAA
TTAGAATTAAAGAATTAAATGTAATAGAAAAGATTATTTGCAAGAAATAAGAAACTGCCATTTTTTTATAGTATTCTG
CCAATTATTCTACTAATAAGAGTATTAAATGTGTAATATTATAACATATATGTAATTTTCTATATGAGTATTTGATCA
TTAAGTTTTCATTTATTTGTGCTTAATTAATATTAATTTTTAAGCAGATAATAATAATGATAAAATGAAAATTCACAAA
ATTAAATTTACTATTTTTTATTAATATTAATATTTAATGCCATTCTCTGCAATATTTTTGCATATTTTTTGCTTTTTCAT
AGAGTGAATAATGATTATAATATGAATATTATGCAATGATTTTTTTTCATCTTGATTTATGTCCGATTTATATTATTAT
ATATTATATATTTATATATTTTTATTATTATATATTATAAATGAAAAAAAAACATGTAGCAAAGTTCTACACAAACATC
ATATTTATCGTATTTAGAAGAAAATCTGTCTTGGTAATTTTCAAATATAATATACTTTTCTTAAAGTATCTTTTAAAAA
TTTTTATCAGAATCTTTATTGAAATTTTTTTATTGTTGTTTGATTAACTATTTTTAAATATTTTAACATATATGTTTGAC
ATAGAAATAGTCTGTATGTGAATTATGTAATTTATTTTGAAAAAATAATTATAAAATTTTATACTATAAATTGTTATTT
TGTTCAAATGATCTACAATTTTCCGTATTTATCAAAAGATTTTTAAACAATATTAGATTTCGTATCATTATTTTAAAAA
ATAATGAGTAATAAATATTCTAAAACATTCAATATTTTTCTTTTCTTAAAGAAGATCTCGCATTTTTATTTTTCTCGTA
TCTTTATTTTTAGTGATAGTTTTTTGATAGAATGTTATAAAATTCATTTCTACTTTCTTTATTGTCGAATCAGACTTTT
ATCTTATTATTTAAATCTCTTTACTTTTGCTGAGCGAATTATGTACATATTAGGTTGCGAGATCATCATCTTAGAAAT
TAATGTAAAGTAAATCCGAGAATGTAATAGCTATATTTTTTATATTAAATATTAAGATAACATTGTCATTTTGAAAGA
AACAATGAAAGTGTCTTCAATTAACATGCCGTACTTGGTTTATAATTATTAGCTAAAACAAGTTATCAGTATAAACA
AGAAAAACAATAGTAATAGTAATAGTAATAGGAAAAAGACAAAAATAAAATAAAAATTCGGGAATCTGCGCCATCT
TTAGAGCCGTAGATTCAACTTAAATTTAAACAAAAAAAATAAGAAAAGGATAAACATAAAGTAAGAATTGATTAATA
GCGCTATCTTGAATATTTTTCCTAAATCATTTAAGAGATGGTATTAATTTCTAATTTTTACTCTACTTTATCTTGCTT
TTAAAGAAATGTCTCTAGCATTCGAGTGATGGCGTTAACAGTCAATTTTTATATTATCTCTGTTTTTCTCCCATTAT
TTTCTGTCCTTTCTTTTTGTGTTTCATTTGCGGCATTCTGAAGATGGCGCTGATTTTAATATTTTTATCCTATCTCTA
TCCTTTTTCTATTATTTGCTTTAGTAATATCTAATAAAAGTAATATCTATATTTAAGTTTCATCTGGCATCTATAATGC
TGATTTTCGAATTTTTATCATTTTTTTTTACTTTATTATATTTTTGATTATATTTATTTTGCAAAATTTATATGTTAAAA
AAACTTCGATTTGATTTTAATTTTGTTTTTTTTTTTTGTTTAGTATATTAATTAAAAATTTTAATAAAATATATTTTATCT
AATCATCTTATATCTCATCGTATCTGTGATAGTTTGAAGAATATTTTTTTTTATTTATCGATAACTGAAAACATCAACA
AAAATTTTATTTTTTCCTATTGTATAGCATTTTTTATCACATTTTGCTGTTTATGAATCTTTATCAATAATTTCAAGCA
GAATTTCTAGTTGAGAAAGAAATTACATCATGCTAAGTTCATGGAACT<u>**TGAAAATCT**</u>TATATATTGTATGGATCCA
TATTCATTTCTCAGTGTCATTCTGTTACAGTGTACGATCACATTGTCAATTATAAATTTGTTTCAATAGTTTCACGA
GAATCAAAATTATTAAATAATTTTTTTATACTTAAAATTCTTTCGACAAGTATAATTTTTTTGTTTTTTACTTTCATCAT
CAAGAAACAACAGATTATAATTCAGAATATTCAAAAGGTAATATTTTATATTTATTAAAAATAGAATTATTTCTTTAT
ATTTCGTATTTTAAATATTTAAGTATATATATATATCATGGATTTTTTTTATTAGATCATAAAGTAGAAGGTAAAGGA
AATAATTCACGAAATATTTAATAGTTATAATTATTAATATAAAAATATAATGAAAATAGTAATGGAAAATTTATTTTTG
TATAAAATTTAATAAAAAATTAATTGACCAAAATTCTGTAAATATTTGGAATGAATAAAATTAAAAAAGAAGATTATA
AAGAATATAAATATTTTAATTATAATAAATAATAAATCTATATTAAAAAATATAATATATCTAATATAATATCTTCATAT
TATTTTATATTAAATTTAAATTGCGTTATAGTATATACTGACGCAATAGTTTTCCGATAATTTGTCACATTGTCGTAA
TAAATTGGTATGATGATATATCGTATAATAATAGAAGACTATATAGTAATTCTATATTATATTATCTATATATATATAT
ATATATATTAATTCTAAGAAGATATACATTAATTAGTTTATATTATTTTTATCATATCCATTTTATAAATATATTTTTTT
TTTAATTAAAAACTTAATTGCTTATCATATATCGTCAAATCATCATAACGTAATAAAACTTCGTATCATTATTATTTAT
CGTAATGTAATAAAACTTCGTATCATTATTAATTTTTTAGAAATTAATAAATAATTAATAGTTTAATAGGTTACAACT
AACATAAATATTAAACCGAAAATAACCTAAATAGATTTCATCGTTTTTTGCTTCTTGCAAAAACGAAATTTGGAAAT
AAAAAGAAAATT

M. rotundata

Gnmt1 gene

TCGGGTAATATTTGCCACCTTGCTCACCTATTTTGTAGGTTGGGTATATGTCGGAGTAATCTCGAAGAAGAGAAA
GTTCGTCCTTAATAATAGGCTACAAAAAAATAAAGAATCGAAAATCGTGCAGTTAAATGAAGATGTCAGCCTTCTG
TTACAAGGAACATCGCATATAAATTTTATTTTTTATTTTATATATATTTATTATATACGATACCAATAATATTTAAGTG

Journal of Experimental Biology • Supplementary information

ACTGAAGCTTTAGAAGGTGCCAATTTAACTCTACAGAGGCTTTATTGTATCCATTTTTATTTCCACACAATATATTC
ATATAAGATCAAAGATTCAAATGATTTACGTAACTATTTCAGAATGGCCCGTGGACAACAAAAGATTCAGTCTCAA
GCTAAAGCAGCAGAAAAAGCTGCGAAAGTAAAGAAACAACAAGGACACAGTGCCAACGATCAAAAAAAAGCTGC
ACAGAAAGCTTTAGTACACGTATGCGTAGTTTGTAAGGTCAGTGAACTTTTGTGAAAGATTCCCTAACATTTATAA
ATTGTTTGCAACTTTTATGTAATCGGGAGGTTCAATGAAAATGGAGCACATACCATGTTCCTTTTGTAACAATTTG
GCAAAAAGAAATTAATTTCTCTGTAAATTTACAGTATTGTGGTATGTAGCACTTTTCTAATGAACTTTTCAGTTCAA
AAATTTGATGTTTATTTGGTAAATATATTTAAGATGCATTTGATAATAAATGTTTTGTACTAATAGGCCCAAATGCC
AGATCCAAAGACTTATAAGCAACATTTTGAGAACAAACATCCCAAGAATGAGTTGCCAGAGGACTTAAAAAATATA
TGAGGGTGCGATTTTTTGTACGATTATGGAGCTGGAATGGAGGAGAAAAGAATTTACTATAAATGACAAAGTGAT
ATACTTCAATGTTCAACGGATCATTACATAGTCTTAAAACAGGAAATAAATGAAACTTTGTTAGCGTTTAAAGCGA
ACTGTTGCACGGTTAATGAATGAATTTTTGTGCACTGTCCCAAATTATTATAACATTATCGATGGCTCGCTTATCG
AATCAAATCTCCATTTTTTCCAAAGATATTCAGCATTCTTCGTCTAATAATATGATTCTTAAGTTTTCACTTAAGCAT
AAAAGCAATTTAAAGATGTATATTGCTAAGAAATAAAGATAATGAATTTAGCAGAATTGTTGCAAGTATTTGTACGT
ATTTTAATGTTGTAGGCAGTAAAATGGTACTCGTTTTTATCCGATTAGAAAATCACATTAATTCAATGTAGGTAGTC
ACGCGATTCTCGAGAGTTTAATAATTGCAAGTAACGATGGAAATGATATCTTACAAGAATTAAGAAACTGCCATTT
TATAGTATCTGCCATTTACTTATTAATGATATTCTATGATTAGAATGCTGTATGACGCGGTGGTCTATAAATATTTG
ATCATTATTTTCGTTTGTATTCGAGCCTAATTTTTATTAAATTTACAGTATTCGCTATTTTGAAATACGTTATATTAAT
ATGGAAATTAATAGATGTTTTTAAGAAAACTGCGTTTTAGTTTTCAGTACTTAAACCCTTCTTTTATACTGAGAATA
GTCGATTCGGTTTTGCCTAATCACAAGAAGTGTTACGCTACGTTGTACAATTATCTTTGTTAAGGAAAATATCAAA
GTATTAAATATAATCAGTATTTATTAGAGAATCTGTTATTCTCAGTAACTAATACATCGTATTTTAAAATATGCTTCG
CCAAACGCATAGAGCTACATCGTGAACATACGTTAACGGTGTTCTGTACGGACATGATTTATAGGAATTCCTAGT
ATGTGTTTAATTTAGGAATAAGTATGCGCATTATGTAATTTATTCTGATAAAATAATTATACAATTTTTCACTACAGT
TGCATCACATTAATCATTTTCCTCATGAGAACTGCAAAATATATGTATCTTGTCGATATCTGTACATCCGGTGATTT
TATTTTTAAAGATACTTTG**TTTCGCGGTA**AGATAAAGAATAAACAGAAAGTGTAATGATAATGATGTCACGTAGTA
GCAGGAAAAATTGCATTACAGAAAACAGTCATTCTTTACTAGTAGATATCATAATATTTTCTCTACGATTTTGCTTT
GTCATGATTGTTTATCAGCCGATTTAAACTGTGCCTAGAAAAACCTACGAAGGATTACATCATTCAGGGATTCAG
GGAGGAATACGGGATCTTATATATGGGTGAATGATCCGCAACTATCTCAGTTTCAAGTTGTCACTGCACGCGGCA
GCGTCGTTCTTTCATTGTCCTAAGTTTTGTGTTTTACAATTTTATAATTCTTGAATGTTTTAATGACTTATCTATAAC
ATTTTATACATGAAAATTCTGTACAAAATCTATCACATTTTCACGGGGTAACAAGTTAAATCAGGTAACGACTTTTT
AAAAATAATTAAATATATTTATTGATAAATTATATCGATCTGTAAATCTGTAATATAAAATTGTTGAGAAGTTTGATA
ATCAAAATCTGGCTTTTATAAAATGACGTAAATGCAACAATCATTATCTTTGTTACAATCAGGTGCACTTGAGTACA
TTATATTGAAAAACAATGACGAAAATTTGTGTAACTGACGAAACACCATATTCGAGGTATCGCTAATCCATTAGTT
TTCTTCGGCTCCTCTTAAAAGTTTCGTAACAGATCTTCTATAATCTAATCACAAACGTACGTTGATCATTTAAATAA
CAGATAAGGGAATAAACATACACATATATACATTTAATTAATCGGGCTTAGTTCTTTGTGAAAAGTATGCCAAACA
TTTGGCAATTTTATGTAACAATTTCAACCGGATCGAATTCTTGAATTCCTGTCGGAATTTGTAGAGTTGAATCTCAC
TTTTCTGCAGGGTTTTTGAAAGCATTAGGCTTCTCCCTATAACAAAAGAAATCGAACTATAATCGCACTAATTTTCA
CATAATAATTTCAGCAAAGTTCGAAAAAGAAATGATGGACTCTGTATTTCGTACCCGCTCGCTCGGTACCGCGGC
AGAAGGTGTCCGCGACCAGTACGCGGACGGAAGAGCC

Journal of Experimental Biology • Supplementary information

(B)

A. mellifera

Gnmt1 mRNA

TGAAAATCTTATATATTGT**ATG**GATCCATATTCATTTCTCAGTGTCATTCTGTTACAGTGTACGATCACATTGTCAA
TTATAAATTTCAATAGTTTCAATAGTTTCACGAGAATCAAAATTATTAAATAATTTTTTTATACTTAAAATTCTTTCGA
CAAGTATAATTTTTTTGTTTTTTACTTTCATCATCAAGAAACAACAGATTATAATTCAGAATATTCAAAAGAAACTAA
CAGGATA**ATGGATTCGATATTTCGTACGAGATCGCTCGGTACGGCAGCCGAGGGCGTTCGAGATCAGTATGCA**
GATGGTAGAGCTGCTAAGGTGTGGGAAGTGTTCATTGGCGACAAAAAGCAGAGAACTCAAAATTACAGAGAC
TTCTTAGTTGGTCTATTGAGAAATAAAGGATGCAAAAGGATTTTAGATGTGGCATGCGGGACTGGAGTAGACT
CGGTGATGCTTCTTGAAGAAGGTTTCGAGGTAGTCAGTGTTGACGCTTCGGATAAAATGCTGAAATATGCTTTG
AAAGCTAGATGGGAACGTCGAAAAGAGCAAGCGTTCGATAACTGGGTAATCGAAGAAGCAAATTGGTTGACT
CTACCAAAAGACATTCATTATTTGTTAAGAGATGGATTTGACGCGGTAATCTGCTTGGGAAACAGTTTTGCTCA
CATGCCTGATACGTTTGGCGATCAACGAGAGCAGAGGCAAGCGTTGTTAAATTTTGAACGTTGTGTGAAACCT
GGTGGTTTATTATTAATCGATCACAGAAATTATGATTTCATTATCGAGACTGGTAATATACCTTCAAAGTGTATA
TATTATAACAGTCAACATATGATAGATATTAAGGCGTCGGTATTATTCGTTTCTGGAAAACCTGCAATTGTGAC
TTTAGATTACATCATTACGATGAACGAAGGAGAAGAGGAAGATGAACAAAAGCAAATCAGTGAATTCAGATTG
TCTTATTATCCTCATAAACTTAGCGTATTCACGGAGATATTGGACGAAGCATTTCATTATAGAGCCAAGCATAC
TATATACGGGGATTTTAAAAATTTGGAAGAAATAAAAAATGCTGGTTTCTATATACATGTGATGGAAAAGCCGA
TATAAGTTCATTTGAGAGAATCGTTTTAAAATTTGAAAACTTTGTAAAACTTTAATGCATCTTGATATAAATATTATA
TCTTCTTA

M. rotundata

Gnmt1 mRNA

TTTCGCGGTACAGATAAAGAATAAACAGAAAGTGTA**ATG**ATA**ATG**ATGTCACGTAGTAGCAGGAAAAATTGCATT
ACAGAAAACAGTCATTCTTTACTAGTAGATATCATAATATTTTCTCTACGATTTTGCTTTGTC**ATG**ATTGTTTATCAG
CCGATTTAAACTGTGCCTAGAAAAACCTACGAAGGATTACATCATTCAGGGATTCAGGGAGGAATACGGGATCTT
ATAT**ATG**GGTGA**ATG**ATCCGCAACTATCTCAGTTTCAAGTTGTCACTGCACGCGGCAGCGTCGTTCTTTCATTGT
CCTAAGTTTTGTGTTTTACAATTTTATAATTCTTGA**ATG**TTTTA**ATG**ACTTATCTATAACATTTTATAC**ATG**AAAATTC
TGTACAAAATCTATCACATTTTCACGGGGTAACAAGTTAAATCAGCAAAGTTCGAAAAAGAA**ATGATGGACTCTGT**
ATTTCGTACCCGCTCGCTCGGTACCGCGGCAGAAGGTGTCCGCGACCAGTACGCGGACGGAAGAGCCGCCA
AAGTCTGGGAGGTGTTTATAGGCGACAAAAAACAGAGGACGCAAAATTATCGAGACTTCCTAGTAGGCTTATT
ACGGGAGAAAGGATGCCGTAGAATCCTGGACGTCGCATGCGGTACAGGCGTCGACTCGGTGATGCTCCTCGA
AGAAGGTTTCGAGGTTGTTAGCGTGGATGCTTCGGACAAGATGCTGAAGTATGCGTTGAAATCTAGATGGGAT
CGACGCAAGGAACCTGCGTTTGATAACTGGGTAATTGAAGAGGCAAACTGGTTGACTTTACCAAAGGACATCA
GTCACGAAATAGCGGACGGTTTCGATGCAGTGATCTGTTTGGGCAACAGCTTCGCTCACATGCCTGACAATTT
CGGCGATCAGAGGGAGCAAAGGCAGGCGCTGCTGAATTTTGAGCGTTGCGTGAAACCGGGTGGTTTGTTGCT
AATCGATCATAGAAATTATGATTACATTATTGAGACTGGGAATATACCTCCTAAATGCATTTATTATAACAGCC
AACACATGACAAATATTAAGGCTTCCGTCCTGTTCGTCTCTGGAAAGCCAGCCATTGTTACCTTGGACTACATG
ATCGCCCTAGAAGAAAACGACGACGAAAATCAAGCTGTCAGTGAATTCAGGCTCTCTTATTATCCGCACCGAT
TAAACGTCTTCAGGGATATGTTGGACGAGGCTTTCCACTACAGAGCTAAGCACACAATTTACGCTGACTTCAA
GACCCTCGAAGAAGTCAAACATCCCGGATTTTATATTCATGTGATGGAGAAACCTGTGTAAATACTTTTCC
CGCCAAAATGTGGTCCATCTATCAGGAGCGTCATGGAAGACAAATCGAACAAATATCGATGTTTCAACGCCATAT
TGTATGTCCACGTTTTTCAGAAAGATGAGTTTAAAATCTTTGTATGATTAATATTATACTTTTATTGTATTTATTGTT
ATAAATACGTAAGGGTATTTAAGATAGATTATAAATGTATAAGATAGAGTATATAGAATTATAAAGGACTAAAGAAT
ATATAAATGTGAATATATATATAAGATAAAGCACTAAAGAAAAATATTTTTACAATATTTATAAAGTATATTTGCATA
TAGTATGAGACTAGAGAACTATTAATTTAGACTTCAAAAGTGGACATACAATATTTGCAGCAAGCTATAACAATGA
ACCTGTGCTCAGACCACAATCACGTTATAATTCATCGTTCATTTGTTTATTTATTTGAAAGTATGTAAAGATCCTTA
AACCATGAAGGATTGATTCGCTGTAACATTTTGTATCATGTAACTATTTTATATTAATGAATCTTGCTACTTCA

Journal of Experimental Biology • Supplementary information

Fig. S5. (A) *A. mellifera and M. rotundata Gnmt* genes (-2kb to +1kb relative to transcriptional start site (TSS)). Underlined region represents first 10 nucleotides of the TSS. (B) *A. mellifera and M. rotundata Gnmt* mRNA. Black bolded sequence is the coding region. Red bolded ATG sequences are upstream ORFs.

Table S1. Primer sequences, statistical analysis, gene list of components of the methionine cycle in *Apis mellifera*, and genes involved in the UPR in *Megachile rotundata* and *Apis mellifera*

Available for download at
https://journals.biologists.com/jeb/article-lookup/doi/10.1242/jeb.246894#supplementary-data

Journal of Experimental Biology • Supplementary information
